# Gibberellic acid and nitrogen efficiently protect early seedlings growth stage from salt stress damage in Sorghum

**DOI:** 10.1038/s41598-021-84713-9

**Published:** 2021-03-23

**Authors:** Adam Yousif Adam Ali, Muhi Eldeen Hussien Ibrahim, Guisheng Zhou, Nimir Eltyb Ahmed Nimir, Aboagla Mohammed Ibrahim Elsiddig, Xiurong Jiao, Guanglong Zhu, Ebtehal Gabralla Ibrahim Salih, Mohamed Suliman Eltyeb Suliman Suliman, Safiya Babiker Mustafa Elradi

**Affiliations:** 1 Joint International Research Laboratory of Agriculture and Agri-Product Safety of the Ministry of Education of China, Yangzhou University, Yangzhou, 225009 China; 2 Department of Agronomy, Faculty of Agricultural and Environmental Science, University of Gadarif, Al Qadarif, Sudan; 3 Department of Agronomy, College of Agricultural Studies, Sudan University of Science and Technology, Khartoum, 13311 Sudan; 4 Faculty of Agriculture, University of Khartoum, 11115 Khartoum, Sudan; 5 Faculty of Forestry, University of Khartoum, Khartoum, Sudan

**Keywords:** Gibberellins, Plant sciences, Plant stress responses, Salt

## Abstract

Salinity one of environmental factor that limits the growth and productivity of crops. This research was done to investigate whether GA_3_ (0, 144.3, 288.7 and 577.5 μM) and nitrogen fertilizer (0, 90 and 135 kg N ha^−1^) could mitigate the negative impacts of NaCl (0, 100, and 200 mM NaCl) on emergence percentage, seedling growth and some biochemical parameters. The results showed that high salinity level decreased emergence percentage, seedling growth, relative water content, chlorophyll content (SPAD reading), catalase (CAT) and peroxide (POD), but increased soluble protein content, superoxide dismutase (SOD) activity and malondialdehyde (MDA) content. The SOD activity was decreased by nitrogen. However, the other measurements were increased by nitrogen. The interactive impact between nitrogen and salinity was significant in most parameters except EP, CAT and POD. The seedling length, dry weight, fresh weight, emergence percentage, POD, soluble protein and chlorophyll content were significantly affected by the interaction between GA_3_ and salinity. The GA_3_ and nitrogen application was successful mitigating the adverse effects of salinity. The level of 144.3 and 288.7 μm GA_3_ and the rate of 90 and 135 kg N ha^−1^ were most effective on many of the attributes studied. Our study suggested that GA_3_ and nitrogen could efficiently protect early seedlings growth from salinity damage.

## Introduction

Salinity is an essential environmental stress that affects plant growth and causes limitations of crop production in the desert and semi-desert areas in the world^3,4^. Worldwide, there are about 95 million ha from whole world land, 45 million ha of the irrigated area were affected by salinity. Moreover, due to increment of salinity about 1.5 million ha are become out of production^5^.

Salinity stress can significantly inhibit germination and seedling growth, decrease many physiological processes and ultimately reduce crop productivity by causing osmotic stress and/or toxicity of ions as well as by reducing the uptake of important ions such as calcium and potassium^6^. Crop plants can suffer from salinity stress at all growth stages, but germination and early plant stage are known to be more sensitive for most plant species^7,8^. Salinity stress affect all growth stages, but germination and early seedling stage are known to be more sensitive to salinity, causing significantly inhibited germination and seedling growth and ultimately decreased crop productivity through osmotic stress and ion toxicity such as Na^+^ and Cl^−^, as well as throught reduced absorption of important nutrients such as Ca^+2^ and K^+^^6,9^.

Fertilization is an effective way to supply nutrients for plants, and it is also an important factor to improve the yield and quality of plants^10^. As compared with other nutrients, nitrogen (N) is required most consistently in larger amounts for crop production^11^. N fertilization has a significant impact on plant growth, development, yield components, and quality, and its effective use to can enhance crop yield in agricultural systems^1^. Saline soil is characterized by an imbalance of essential nutrients in the soil and leads to decreased absorption of these elements, especially nitrogen, phosphorus, and potassium in the root system. Previous studies have observed that the relation between salinity and mineral nutrition is very complicated and not well understood^7^. Zhang et al.^12^ reported that the application of fertilizer could balance the nutrients in the cytosol and improve nutrient absorption and utilization. Further, saline soils usually increase osmotic pressure in the root medium and reduce the responses of plant to fertilizer application, which is the main reason for decreased photosynthesis in plant under salinity stress^7^.

Gibberellins is one of the major plant hormones and an efficient and broad-spectrum plant growth regulator. Gibberellins have reported being a promoter for plant growth under salinity conditions, which can reduce seed dormancy, improve plant gene expression, enhance the synthesis of hydrolase, repair injured cell membranes, and increase seed vitality^13^. In current times, many investigations have revealed that the treatment of exogenous gibberellic acid (GA_3_) can significantly increase seed germination, increase the salt tolerance of seeds, and mitigate the inhibition of salt on seedling growth^13,14^.

Sorghum [*Sorghum bicolor* (L.) Moench] is one of the main crops because of its high productivity and high nutritive value. It can adapt to different environmental conditions, especially in arid and semi-arid areas^15^. Sorghum is considered moderately tolerant to soil salinity. Germination and seedling stage of sorghum grown on salinity soils are essential for the final production and yield^16^.

The seedling stage is important for plant life and crop production. For good plant establishment and great production, alleviating ways should be developed to improve early plant growth under abiotic stress. However, to our knowledge, there are little research on the impacts of external GA_3_ application and nitrogen application on seedling characteristics and anti-oxidative defense system of sorghum under salinity conditions. Many reports have focused on the alleviation impacts of gibberellic acid on salinity stress. In this study, we hypothesized that seed pre-soaking by GA_3_, and soil treated by nitrogen fertilizer could improve the crop establishment through increase the seedling emergence, and improve seedling growth characteristics of sorghum. Therefore, this study was done to examine the impacts of nitrogen application, gibberellic acid and sodium chloride on the morphological attitudes and the activities of antioxidant enzyme of sorghum seedling.

## Results

The ANOVA table indicated that salinity, nitrogen, gibberellic acid and their interactions produced different effects on most parameters, including seedling emergence percentage, seedling growth characteristics, relative water content, chlorophyll content (SPAD reading), protein content, the activities of superoxide dismutase (SOD), peroxide (POD), catalase (CAT), and malondialdehyde (MDA) content (Table 1).

**Table 1:** Summary of analysis of variance (ANOVA table) for emergence percentage, seedling length, fresh weight, dry weight, Chlorophyll content (SPAD reding), relative water content (RWC), protein content, superoxide dismutase (SOD), catalase (CAT), peroxide (POD), and malondialdehyde (MDA) of sorghum seedlings as effected by salinity, nitrogen and gibberellic acid and their interactions.

Source of variance	F value										
	Emergence percentage	Seedling length	Fresh weight	Dry weight	RWC	Chlorophyll content	Protein content	SOD	CAT	POD	MDA
Nitrogen (N)	43.0**	148.7***	388.6***	2389.1**	151.2**	127.2**	176.7**	181.1**	9.69*	5.5*	171.3**
Salinity (S)	9.74**	297.89***	97.4**	154.5**	14.3**	392.4**	26.0**	8.2**	1.68*	4.8*	20.1**
N × S	0.52^ns^	2.77*	9.5**	54.0**	3.0*	33.2**	7.2**	5.0**	0.20^ns^	1.5^ns^	1.4*
Gibberellic acid (G)	0.56^ns^	135.63**	3.6*	35.2**	2.1^ ns^	2.3*	63.3^ ns^	15.1**	17.7**	74.4**	135.8**
N × G	2.26*	7.79**	3.0*	210.6*	3.3**	1.2^ ns^	54.4**	3.6*	0.27^ns^	11.8**	9.2^ns^
S × G	2.21*	2.70*	0.96^ns^	23.0**	1.6^ns^	2.9*	4.7**	4.4^ns^	2.6*	3.9**	5.8**
N × S × G	1.09^ns^	1.09^ns^	2.05ns	2.3^ns^	2.3ns	5.2^ns^	0.5^ns^	0.6^ns^	0.5^ns^	1.0^ns^	0.3^ns^

*ns* not significant.

*Significant at the 0.05 probability level.

**Significant at the 0.01 probability level.

***Significant at the 0.001 probability level.

### Emergence percentage

Emergence percentage (EP)  increased with application of nitrogen and gibberellic acid (GA_3_). At the high salinity level of 200 mM NaCl, EP was increased by 11.78% when the plants were treated with 288.7 μM GA_3_ as compared with control (0 μM GA_3_) (Table 2). In the interaction between nitrogen and gibberellic acid, the highest EP (84.44%) value was determined on the interaction between 135 kg N ha^−1^ with 288.7 μM GA_3_, while the lowest emergence percentage value was recorded at the 0 kg N ha^−1^ with 288.7 μM GA_3_. Emergence seedling percentage was reduced with increased NaCl salinity level (Table 3).

**Table 2:** Impacts of interaction between different salinity levels and different gibberellic acid levels on emergence percentage, fresh weight, dry weight, protein content and MDA content on sorghum seedlings.

Salinity (mM NaCl)	Gibberellic acid (μM GA_3_)	Emergence percentage (%)	Fresh weight (g plant^-1^)	Dry weight (mg plant^-1^)	Protein content (mg g^–1^ FW)	MDA (µg g^−1^ FW)
0	0	67.78cd	0.25b	84.90c	16.78e	22.94g
	144.3	80.01a	0.28ab	104.4a	19.79 d	26.37fg
	288.7	75.25b	0.30a	95.14b	17.81de	28.35efg
	577.5	80.56a	0.27ab	69.01d	18.89de	35.15bcd
100	0	65.56d	0.14e	47.84h	21.68d	24.45f
	144.3	73.89bc	0.18de	65.56d	23.62dc	28.08e
	288.7	73.33bc	0.21c	78.77c	24.01c	28.65e
	577.5	71.11c	0.19d	71.22d	20.35d	31.87c
200	0	63.33e	0.08g	36.31g	27.72b	33.41cd
	144.3	66.11d	0.11f	45.92f	25.65c	37.7b
	288.7	70.89c	0.09g	47.35f	32.34a	40.25ab
	577.5	66.67d	0.12f	51.41e	30.23ab	44.71a

Within the same column, means followed by the different letters are statistically different at *P* ≤ 0.05.

**Table 3:** Effects of different nitrogen rates and different gibberellic acid (GA_3_) levels on emergence percentage, seedling length, fresh weight, dry weight, relative water content (RWC) protein content (mg g ^–1^ FW), SOD, and POD of sorghum seedlings.

Nitrogen (kg^-1^ ha^−1^)	Gibberellic acid (μM GA_3_)	Emergence percentage (%)	Seedling length cm)	Fresh weight (g plant^-1^)	Dry weight (mg plant^-1^)	RWC (%)	Protein (mg g^–1^FW)	POD (µg g^−1^ FW)	SOD (µg g^−1^ FW)
0 N	0	79.22b	15.50g	0.070f	19.62h	34.36f	18.68cd	24.98f	10.73f
	144.3	82.22ab	24.32bc	0.096ef	24.14ef	31.28fg	17.74cd	40.53d	13.76e
	288.7	47.78e	23.49c	0.120e	30.04e	32.44fg	17.19 d	28.42 ef	12.91ef
	577.5	77.22bc	25.54b	0.072f	20.74f	32.66fg	17.77cd	31.02e	14.19de
90 N	0	60.21d	16.39f	0.137de	51.70de	96.05b	31.60b	25.94ef	13.90e
	144.3	60.35d	17.48ef	0.155d	49.17de	115.49a	34.64ab	36.41de	17.89d
	288.7	80.56ab	18.79e	0.137de	54.35d	83.08cd	36.50a	37.54de	22.01b
	577.5	58.89de	20.89d	0.209c	48.45e	83.59cd	33.76ab	32.21de	26.58a
135 N	0	73.33c	18.02e	0.268cb	97.73c	74.55e	20.22cd	108.60c	19.53c
	144.3	77.22bc	24.98bc	0.301ab	142.57a	81.73d	18.68cd	85.48bc	21.34bc
	288.7	84.44a	23.73c	0.343a	136.86ab	81.45d	21.16c	155.20b	20.76bc
	577.5	72.78c	28.01a	0.299b	122.44b	86.91c	19.94cd	185.70a	18.74cd

Different letters in the same column show significant differences at the 0.05 probability level.

### Seedling length

**Figure 1:**
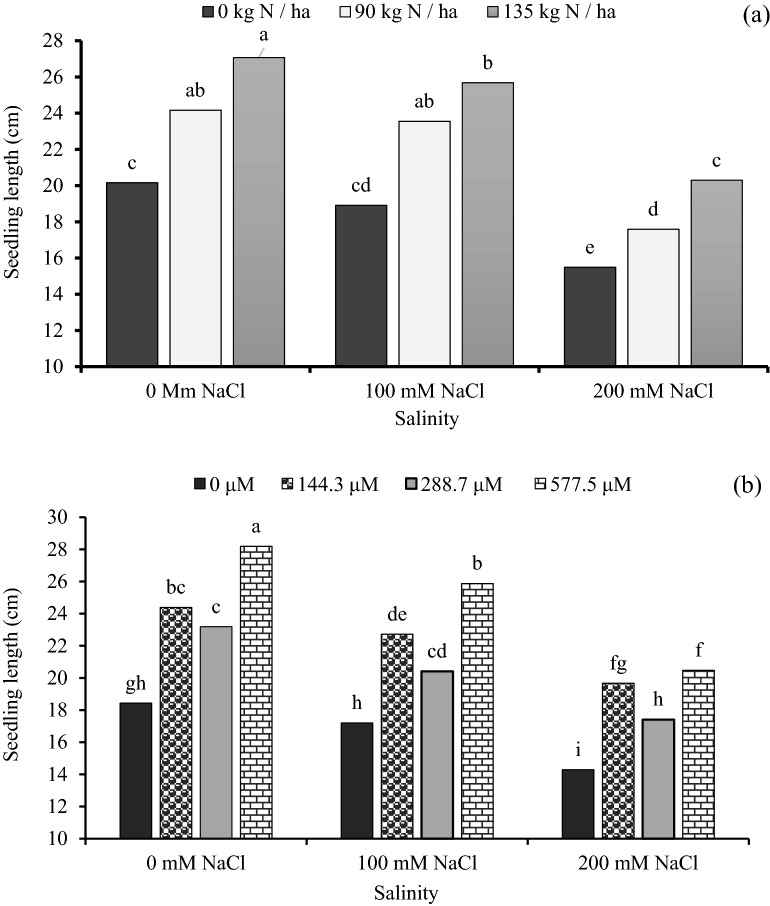
The effect of interaction between: (**a**) different concentrations of salinity × different concentrations of nitrogen and (**b**) different concentrations of salinity × different concentrations of gibberellic acid (GA_3_) on seedling length (cm) of sorghum seedling. Bars with different letters are significantly different at the 0.05 probability. Means were separated by the LSD test.

The nitrogen and GA_3_ treatment affected seedling length positively. At the highest salinity level of 200 mM NaCl, seedling length was increased by 15.4% and 13.6% when the seeds were treated with 135 and 90 kg N ha^−1^ respectively, as compared with 0 mM NaCl (Fig. 1a). However, at the same salinity level, as compared with control of gibberellic acid (0 μM GA_3_), the levels of 577.5 and 144.3 μM GA_3_ increased the seedling length by 53.0% and 32.3% respectively. The seedling length decreased gradually with increased salinity (Fig. 1b). For the interaction between nitrogen and gibberellic acid, the highest seedling length (24.98 cm) value was determined on 135 kg N ha^−1^ with 144.3 μM GA_3_, while the lowest seedling length value recorded at the 0 kg N ha^−1^ with 0 μM GA_3_ (Table 3).

### Fresh weight and dry weight

Fresh weight and dry weight was increased by GA_3_ and application of nitrogen, and decreased with increased salinity level (Table 2). However, at the high salinity level of 200 mM NaCl, as compared with 0 μM GA_3_, 577.5 μM GA_3_ level increased fresh weight and dry weight by 50.0% and 41.6%, respectively (Table 2). Moreover, for the interaction between nitrogen and GA_3_, the highest fresh weight (0.343 g plant^−1^) and dry weight (142.6 mg plant^−1^) value were determined on the 135 kg N ha^−1^ with 288.7 and 135 kg N ha^−1^ with 144.3 μM GA_3_ respectively. While, the lowest fresh weight and dry weight were recorded at the 0 kg N ha^−1^ with 0 kg N ha^−1^ (Table 3). As compared with control of nitrogen, at the high salinity level, the rate of 135 kg N ha^−1^ increased fresh weight and dry weight by 89.5% and 59.1% respectively (Table 4).

**Table 4:** Impacts of salinity and nitrogen on fresh weight, dry weight, relative water content (RWC), CAT, POD, protein content and MDA content on seedling of sorghum.

Salinity (mM NaCl)	Nitrogen (N kg^−1^ ha^−1^)	Fresh weight (g/plant^-1^)	Dry weight (mg/plant^−1^)	RWC (%)	CAT (µg g^−1^ FW)	POD (µg g^−1^ FW**)**	Protein (mg g^–1^ FW)	MDA (µg g^−1^ FW)
0	0	0.20c	37.04d	85.86c	59.24bc	51.89c	13.47g	19.96f
	90	0.29b	83.82ab	101.87a	66.50b	62.08b	16.29c	34.61de
	135	0.36a	94.22a	65.78d	74.11a	77.25a	31.77e	36.99ef
100	0	0.12cd	30.05e	82.72c	48.77d	38.95dc	22.10e	32.47de
	90	0.15d	36.23d	92.78b	36.20e	49.13bc	30.16b	42.77bc
	135	0.34ab	81.27b	59.69d	61.05bc	42.45c	38.12c	53.14e
200	0	0.06e	22.82f	74.91cd	28.53ef	27.90e	17.90b	26.96c
	90	0.10f	32.70de	89.01b	37.21e	33.26d	24.26a	29.41a
	135	0.19c	59.22c	51.76e	48.36d	31.50de	28.07bc	46.93b

Within the same column, means followed by the different letters are statistically different at P ≤ 0.05.

### Chlorophyll content (SPAD reading)

**Figure 2:**
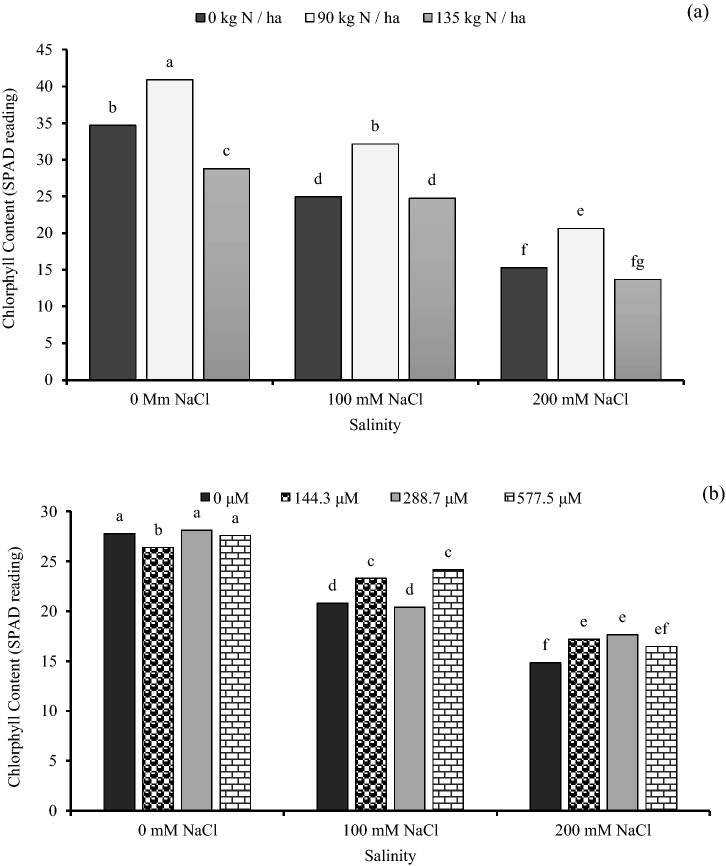
The effect of interaction between: (**a**) different concentrations of salinity × different concentrations of nitrogen and (**b**) different concentrations of salinity × different concentrations of gibberellic acid (GA_3_) on chlorophyll content (SPAD reading). Bars with different letters are significantly different at the 0.05 probability. Means were separated by the LSD test.

The chlorophyll content was decreased with increased salinity level. Chlorophyll content was improved by nitrogen application and GA_3_ amendment. At the high salinity level of 200 mM NaCl, the rate of 90 kg N ha^−1^ increased chlorophyll content by 35.0% as compared with 0 kg N ha^−1^ (Fig. 2a). At the same salinity, the level of 288.7 and 144.3 μM GA_3_ had the highest chlorophyll content (17.62 and 17.19 respectively) (Fig. 2b).

### Relative water content

Relative water content (RWC) was increased with nitrogen treatment and was decreased gradually with increased salinity. The interaction between high salinity level of the 200 mM NaCl with rate of 90 kg N ha^−1^ increased RWC by 21.3% as compared with 200 mM NaCl plus 0 kg N ha^−1^ (Table 2). In the interaction between nitrogen and GA_3_, the highest value of RWC (115.5%) recorded at 90 kg N ha^−1^ with 144.3 μM GA_3_. Moreover, 288.7 μM GA_3_ with 0 kg N ha^−1^ recorded the lowest value of RWC (31.3%) (Table 4).

### Catalase and peroxidase activities

**Figure 3:**
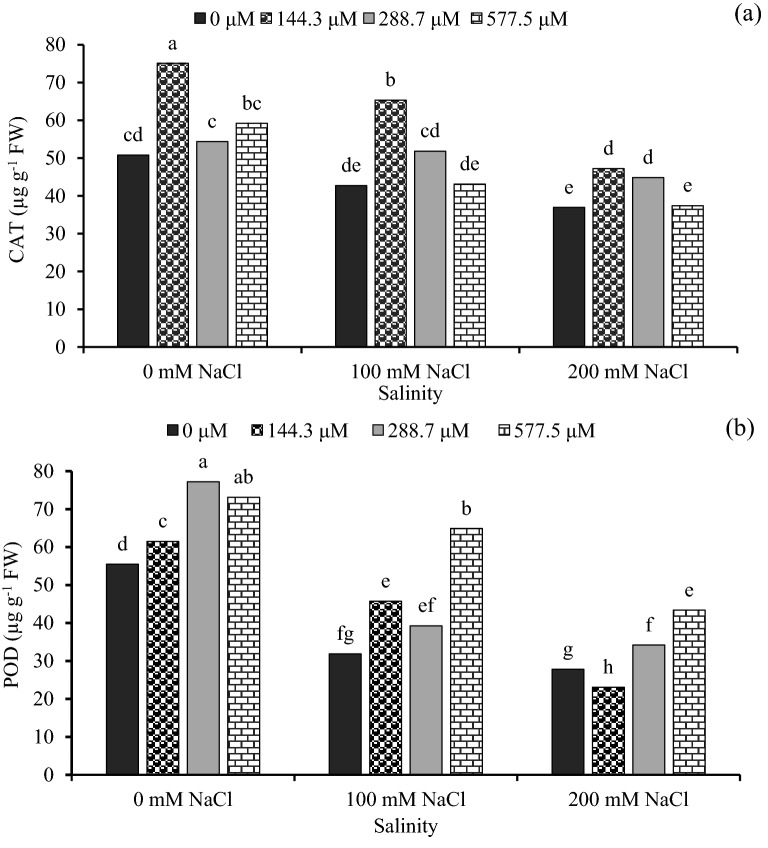
The effect of interaction between different concentrations of salinity × different concentrations of gibberellic acid (GA_3_) on (**a**) catalase (CAT) and (**b**) and peroxidase (POD) activities of sorghum seedling. Bars with different letters are significantly different at the 0.05 probability. Means were separated by the LSD test.

The catalase (CAT) and peroxidase (POD) activities were enhanced by nitrogen application and exogenous GA_3_, but decreased by increased salinity level. In the interaction between salinity and GA_3_, at the 200 mM NaCl, the highest value of CAT and POD activities values were recorded at the 144.3 μM GA_3_ (Fig. 3a) and 577.5 μM GA_3_ (Fig. 3b) respectively. in the interaction between nitrogen and GA_3_, the highest value of POD activity (185.7 µg g^−1^ FW) was observed at 135 kg N ha^−1^ with 577.5 μM GA_3_, while the lowest POD (25.0 µg g^−1^ FW) activity value showed at 0 μM GA_3_ plus 0 kg N ha^−1^ (Table 3). At the high salinity level of 200 mM NaCl, the N rates of 135 and 90 kg N ha^−1^ increased CAT and POD activity by 69.5% and 22.2% respectively as compared with 0 kg N ha^−1^ (Table 4).

### Superoxide dismutase

**Figure 4:**
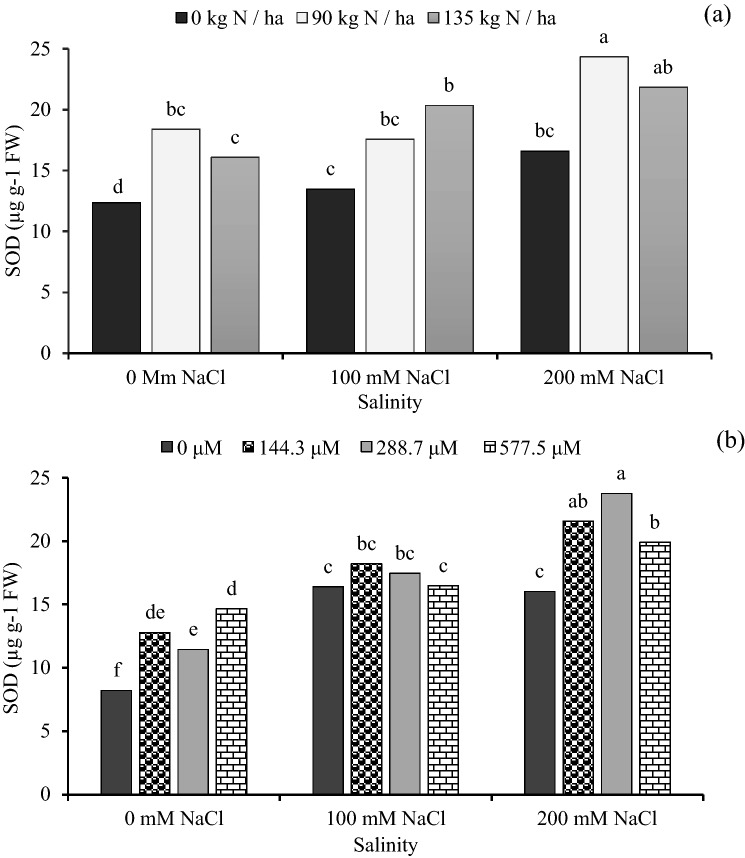
The effect of interaction between: (**a**) different concentrations of salinity × different concentrations of nitrogen and (**b**) different concentrations of salinity × different concentrations of gibberellic acid (GA_3_) on SOD of sorghum seedling. Bars with different letters are significantly different at the 0.05 probability. Means were separated by the LSD test.

Superoxide dismutase activity (SOD) was increased by salinity, nitrogen and GA_3_. The highest SOD activity value was recorded at the 200 mM NaCl with 90 kg N ha^-1^, however the lowest SOD activity was recorded at 0 mM NaCl plus 0 kg N ha^-1^ (Fig. 4a). In addition, the highest activity of SOD (23.77 µg g^-1^ FW) was recorded at 288.7 μM + 200 mM NaCl (Fig. 4b). Seeds treated with 90 kg N ha^-1^ plus 577.5 μM GA_3_ increased SOD activity by 147.7% as compared with 0 kg N ha^-1^ with 0 μM GA_3_ (Table 3).

### Malondialdehyde and soluble protein content

The malondialdehyde content (MDA) and soluble protein content were increased with salinity, nitrogen rates and GA_3_ levels. The highest MDA content (44.71 µg g^−1^ FW) and soluble protein content (32.3 mg g^–1^ FW) were recorded at 200 mM NaCl plus 577.5 μM GA_3_ and 200 mM NaCl + 288.7 μM GA_3_, respectively (Table 2). Moreover, the highest soluble protein content (36.5 mg g^–1^ FW) was recorded at 577 μM plus 90 kg N ha^−1^ (Table 3). As compared with the 0 mM NaCl + 0 kg N ha^−1^, the rate of 90 kg N ha^−1^ plus 200 mM NaCl increased soluble protein content and MDA content by 20.0% and 43.7%, respectively (Table 4).

## Discussion

High salinity stress affects plant growth by changing their physiological parameters^1^. In this study, seedling emergence percentage and seedling growth (root and shoot length, fresh and dry weight) and relative water content were decreased with increased salinity. The reductions in emergence percentage might be due to the decrease in water uptake and enzyme activity caused by salinity^17^. Similar effects were shown in sweet sorghum^17^, wheat^18^, and forage sorghum^19^. In the present study, shoot and root length decreased as salinity level increased. The decline in root and shoot length and development might be due to ions toxicity or reduction in osmotic potential caused by decreased water uptake and nutrient absorption. The impacts of higher salinity on the root growth were more pronounced than shoot growth and caused the reductions in seedlings growth^20,21^. Some studies indicated that reduced growth of the plant due to the proportional increase of sodium^21^. Our results are in agreement with Khan et al.^22^, Nimir et al.^17^ and Ibrahim et al.^3^. The reduced in fresh and dry weight at the salinity stress might be due to the lowest water absorption cause by physiological drought^23,24^. Our findings are in agreement with those of Dheeba et al.^25^, who reported that salinity reduced the fresh and dry weight of plants. Ibrahim et al.^2^ noted that the negative correlation between growth characteristics and salinity stress. The result of decreased in RWC under salinity stress similar with the findings of Ibrahim et al.^21^.

In this investigation, nitrogen application increased seedling emergence percentage, seedling growth and RWC, and alleviated the negative effects of salinity. The increase in emergence percentage by introgen is in agreement with Ibrahim et al.^3^ who reported that the lowest emergence percentage was achieved at the high salinity with the control of nitrogen. However, these findings are in disagreement with those of Fallahi and Khajeh^26^, who found that nirogen application had a negative effect on seed germination and emergence percentage under saline stress. The increase in seedling growth under salinity stress by nitrogen is in agreement with Ibrahim et al.^3^ and Xiong et al.^27^, who reported that nitrogen fertilizer significantly increased root and shoot length, fresh and dry weight under soil salinity conditions.

In the present study, GA_3_ treatment enhanced seedling emergence percentage, seedling growth and RWC. GA_3_ significantly affected emergence percentage, and alleviated the adverse impact of salinity stress by improving water uptake and increase cellular membrane plasticity^28^, which can stimulate the activity of amylase in cotyledons and the conversion of insoluble starch into soluble sugars for seed germination and promote radical growth^29,30^. Similar results of increased emergence percentage and seedlings length, by GA_3_ were reported by Chauhan et al.^24^. However, our results were different from those of Chen et al.^31^ and Shaddad et al.^32^, who reported that GA_3_ had negative effects on shoot and root length in wheat and soybean (*Glycine max*) plants. The difference between these two studies probably lies in the differences in crop species and the levels of GA_3_. Shaddad et al.^32^ reported that the exogenous gibberellic acid can improve the growth and physiological parameters of the plant, which are responsible for increased fresh and dry weight of the plant.

In this study, GA_3_ treatment also improved relative water content (RWC). These results were in agreement with those of Ghodrat and Rousta^33^ and Chauhan et al.^24^, who reported that the RWC was increased in response to GA_3_ application. Soaking seeds with suitable levels of GA_3_ plays an important role in the induction of develop salinity tolerance such as; selective accumulation and/or exclusion of ions, ion uptake control by roots and transport into leaves, compartmentalization of ions at the cellular and whole plant levels, synthesis of compatible solutes, change in photosynthetic pathways, alteration in membrane structure, induction of antioxidative enzymes and induction of some plant hormones^34^. Also, overcome limitations created by the environmental stress such as nutritional imbalance, osmotic effects and ion toxicity increases salinity tolerance in the crops^19^. In the study, nitrogen treatment also increased RWC in the leaves of seedlings sorghum under salinity stress. The type of nitrogen sources or nitrogen level may cause the increased in RWC.

Increased in the anti-oxidative enzymes under salt stress could be suggestive of an increased of ROS and improvement of a protective mechanism to decrease oxidative harm triggered by stress in plants. In this study, salinity stress caused a significantly increased in the soluble protein content, malondialdehyde content (MDA), superoxide dismutase activity (SOD). While, catalase (CAT) and peroxidase (POD) activities was decreased with salinity levels increased. This increased in antioxidant enzyme activity might be due to the activation of plant resistance mechanisms^1^. The increased of accumulation protein may be due to rapid accumulation of a specific set of protein in plant^14^. This results are in disagreement with those of Abdoli and Shekafandeh^35^, Bano et al.^36^, Ibrahim et al.^7^, and Hatami et al.^37^. The decreased in protein content under salinity stress may be due to increased sodium content and consequently reduction in potassium concentration in the cell. Increase in SOD activity could increase the ability of the seedlings to scavenge O_2_ and remove the accumulation of ROS, which could cause a reduction the membrane damage. An increased of protein content and SOD activity by salinity were also reported by Ali et al.^19^, Zrig et al.^38^, and Nimir et al.^17^. On other hand, Dissimilar result was reported by Qiu et al.^39^ in wheat plant, and Sekmen et al.^40^ in *Gypsophila oblanceolate*, who mentioned that SOD, CAT and POD activities were decreased with increased salinity. The difference results between these two studies probably in the difference in crop species. This results were similar with those of Ali et al.^19^, and Ibrahim et al.^7^, who observed that salinity stress increased MDA, CAT and POD activities. In this study, salinity stress inhibited chlorophyll content as SPAD reading. The decreased in chlorophyll content under salinity stress because of the inhibitory effects of ions of several salts on the biosynthesis of different chlorophyll molecules^41^. These results were in accordance with Jamil and Rha^42^.

In this study, we observed that chlorophyll content (SPAD reading) and antioxidant enzymes activity (SOD, and POD) were decreased slightly with increased nitrogen rate. Related findings have been observed by those of Huang et al.^43^ and Ibrahim et al.^7^. In this study, CAT was initially increased with increased nitrogen rate. Related increase in the CAT activity have been also observed by those of Ibrahim et al.^7^, and Huang et al.^43^. Moreover, MDA and protein content were increased when the plants were treated with N. These findings are in agreement with Ibrahim et al.^7^.

In this study, the chlorophyll content, soluble protein, MDA content and activity of antioxidant enzymes were increased with increased GA_3_. Our study explained that exogenous GA_3_ could increase SOD, CAT and POD activities in sorghum seedlings under salt stress, and improved the seedlings ability to combat oxidative damage. This observation was contrary to the findings of Ali et al.^19^ and Zhu et al.^44^, who reported that SOD activity in salt stressed was decreased by exogenous application of hormones. Similar result was reported by Zhu et al.^44^ who reported that CAT and POD activities improved by GA_3_ amendment was beneficial for okra plants to be more efficient in breaking H_2_O_2_ in to O_2_ and H_2_O. While, Tuna et al.^45^ reported that POD activity were decreased by increased exogenous GA_3_ at 144.3 and 288.7 µM in maize plant under. The difference results in POD activity between these two studies probably lies in the difference in crop species and their activity to GA_3_ concentration^46^. Related increased in MDA content have been also observed by those of Ali et al.^8^, who reported that MDA content was increased with increased hormone.

## Conclusion

Nitrogen was beneficial or even necessary for sorghum growth and development, especially under saline conditions. Our study investigated the effects of external application of GA_3_ and nitrogen application on seedlings emergence percentage, seedlings growth and antioxidant enzymes of sorghum seedlings subjected to salinity. The findings from this study showed that seedling emergence percentage, seedling growth, and antioxidant enzymes were inhibited by NaCl salinity stress**.** Nitrogen and GA_3_ had a positive effect on seedlings emergence percentage; seedling growth and antioxidant enzymes by increased these parameters. However, from the present study, it can be concluded that nitrogen management is important when the plant growth in the salinity soil. Further, study to examine the effect of nitrogen and GA_3_ is needed to optimize the effectiveness of nitrogen fertilizer and seed treatments with GA_3_ on more cultivars of sorghum will help us to see if there is any relationship between nitrogen and GA_3_ and salinity tolerance of the seeds during seedling growth stages.

## Materials and methods

### Experimental site and soil characteristics

A controlled pot experiment was carried two times in a growth chamber at Joint International Research Laboratory of Agriculture and Agri-Product Safety of Ministry of Education of China, Yangzhou University, Yangzhou, Jiangsu Province, during 2019, to examine the impacts of GA3 and nitrogen on the emergence percentage, morphological attributes and antioxidant enzyme of the sorghum seedling under salinity.

### Plant materials

Sorghum [*Sorghum bicolor* (L.) Moench] seeds obtained from the Agricultural Research Institute, Sudan were used in the research. Seeds were selected for color, shape, and symmetric size. Before the study, the sorghum seeds were surface-sterilized with 1% sodium hypochlorite solution for 2 min and then were washed three times with distilled water, and then was dried with air to their original weight (50 g).

### Preparation of treatment and experimental design

This study contented three factors, including three salinity concentrations at 0, 100 and 200 mM NaCl, three rates of nitrogen (0, 90 and 135 kg N ha^−1^), and four gibberellic acid levels were applied: 0, 144.3, 288.7 and 577.5 μM. The study was conducted in an RCBD as a factorial experiment arranged in a split–split-plot with three replications. The main plots included three different salinity levels, the subplots included three different levels of nitrogen fertilizer as urea and the sub–sub-plot included four levels of gibberellic acid. Before seed planting, seeds (50 g) were soaked in 500 mL of one of the gibberellic acid solutions under dark conditions for 12 h, while the control was treated with distilled water. The seeds were dried with forced air for 48 h to their original weight to safe moisture content of seeds. The pots used in this study are 9.5 cm in diameter and 8.5 cm in depth. Each pot was filled with 400 g washed sand. Nitrogen treatment was made by nitrogen solution at the first irrigation with the same amount (10 mL) of 0, 90 and 135 kg N ha^−1^. Urea is the source of nitrogen fertilizer. Salinity treatment was made by the NaCl solution in each pot by the first irrigation with the same amount (80 mL)^2^. Ten seeds were sown in each pot at 2 cm in depth. All the pots were placed in the growth chamber (Model PYX-300G-B, Yangzhou Yiwei Automatic Instrument Co. Ltd, Jiangsu, China) for three weeks at 30/25 °C day/night. The relative humidity was maintained at 55–60% and 14/10 h day/ night under a photoactive radiation (PAR) of 500 W m^−2^^21,47^^.^

### Measurements

#### Emergence percentage (EP%)

Seedlings were considered emerged when the coleoptiles were visible above the substratum surface. After 10 days, seedling emergence percentage was calculated with the following formula:

$$\mathrm{EP\%}=\frac{\mathrm{No}. \; \mathrm{ of \; emerged \; seedlings \; after }\; 10\; \mathrm{ days}} {\mathrm{Total \; No}.\; \mathrm{ of \; seeds \; on \; pot}}\times 100$$

#### Seedlings growth attributes

At 21 days after planting, the seedlings of each pot were harvested and washed. Seedling growth parameters were measured including seedling length, fresh weight (FW) and dry weight (DW). DW was recorded after dry the seedlings in the oven at 80 °C for 80 h.

#### Relative water content

Relative water content (RWC) was measured according to the described by Mäkelä et al.^48^. Leaflet samples were harvested from the three plants. The FW was determined, and the leaves were kept in water for 8 h for saturation weight measurement. The samples were dried in a hot air oven at 80 °C for 72 h to determine DW. The RWC was calculated as the following formula:

$$\mathrm{RWC}=\frac{[\mathrm{Fresh \; weight}-\mathrm{Dry \; weight}]}{[\mathrm{ Saturation \; weight}-\mathrm{Dry \; weight}]}\times 100$$

#### Chlorophyll content (SPAD reading)

Eighteen days after planting, the penultimate leaves of each seedling in each pot were used for SPAD determination with a chlorophyll meter (SPAD-502, chlorophyll meter, Minolta Camera Co., Ltd., Japan). The SPAD reading was recorded for three seedlings. The average of SPAD readings of the seedlings of each pot was calculated.

#### Determination of biochemical attribute

The soluble protein content was determined according to Bradford^49^. The peroxide (POD) activity was assayed according to the method of Xu and Ye^50^. The activity of superoxide dismutase (SOD) and catalase (CAT) was measured following the method of Janmohammadi et al.^51^. The malondialdehyde (MDA) content was determined following the method of Zhang et al.^52^.

### Statistical analysis

The data of each variable were statistically analyzed of variance for RCBD as a factorial design with the statistical package of MSTATC^53^. When F values were significant, means were separated by the least significant difference (LSD) test (P ≤ 0.05 probability) as described by Snedecor and Cochran^54^.
